# Supplementary figures and images for: A high-resolution two-step evolution experiment in yeast reveals a shift from pleiotropic to modular adaptation
Source: PLoS Biol. 2024 Dec 5;22(12):e3002848. doi: 10.1371/journal.pbio.3002848 (PMC11620474; doi:10.1371/journal.pbio.3002848)

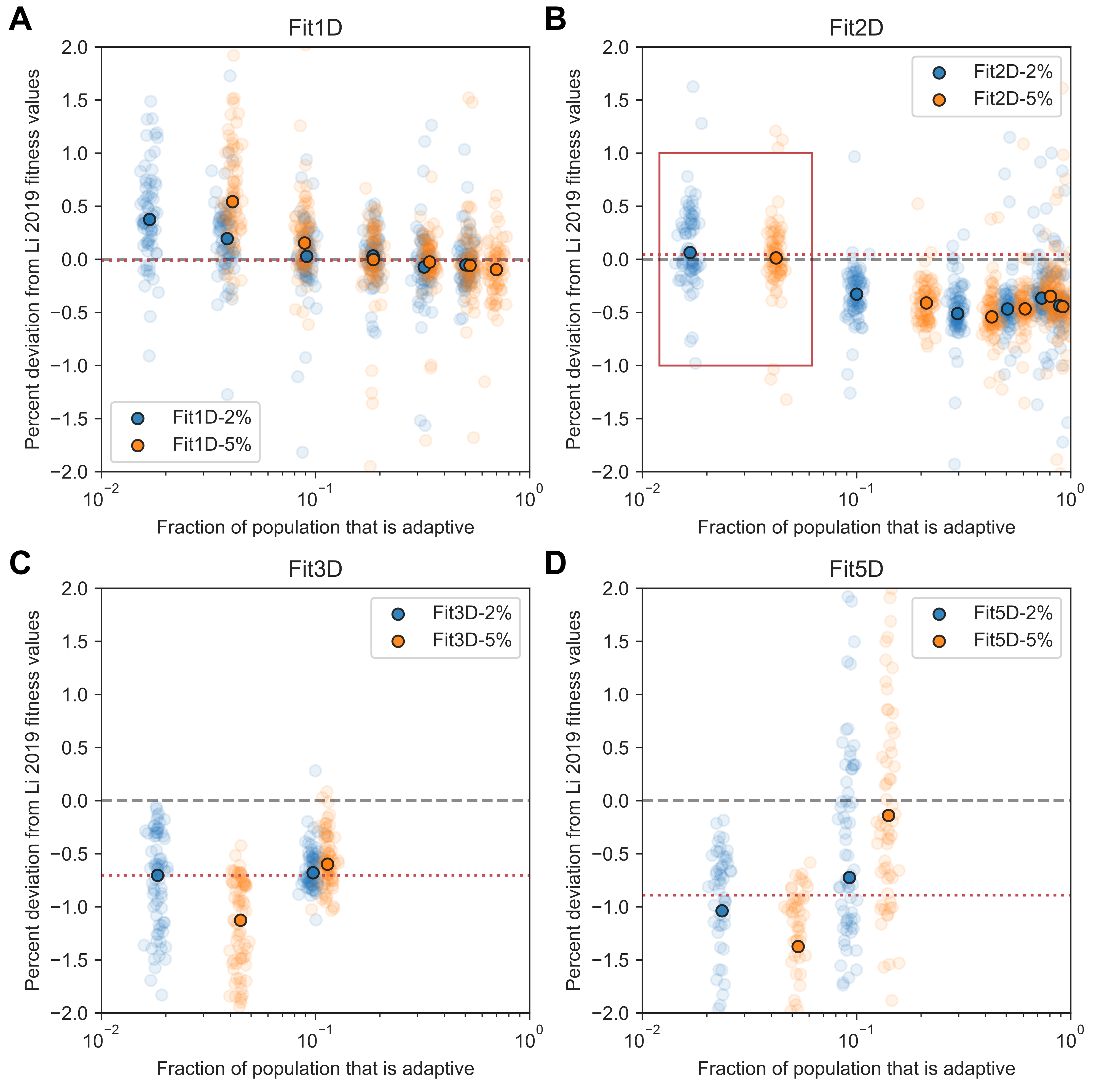

Supplement: S1 Fig — The vertical axis of each subplot depicts the percent deviation from Li 2019 fitness values for the set of adaptive haploids that were present in Li 2019 fitness measurements and this study. The horizontal axis is the fraction of the population that is adaptive. Points show the deviation for each mutant, with the median across all mutants depicted by the heavy circle. Blue and orange points are from experiments initiated with the adaptive barcode pool consisting of 2% and 5% of the population, respectively. Red dotted line indicates the deviation for the overall fitness measurement used throughout the paper. Red box in (B) refers to the time points used. Subpanels A–D refer to Fit1D, Fit2D, Fit3D, and Fit5D fitness values, respectively. The data and code underlying this figure can be found in https://zenodo.org/records/13336585. (TIF) [file pbio.3002848.s001.tif]

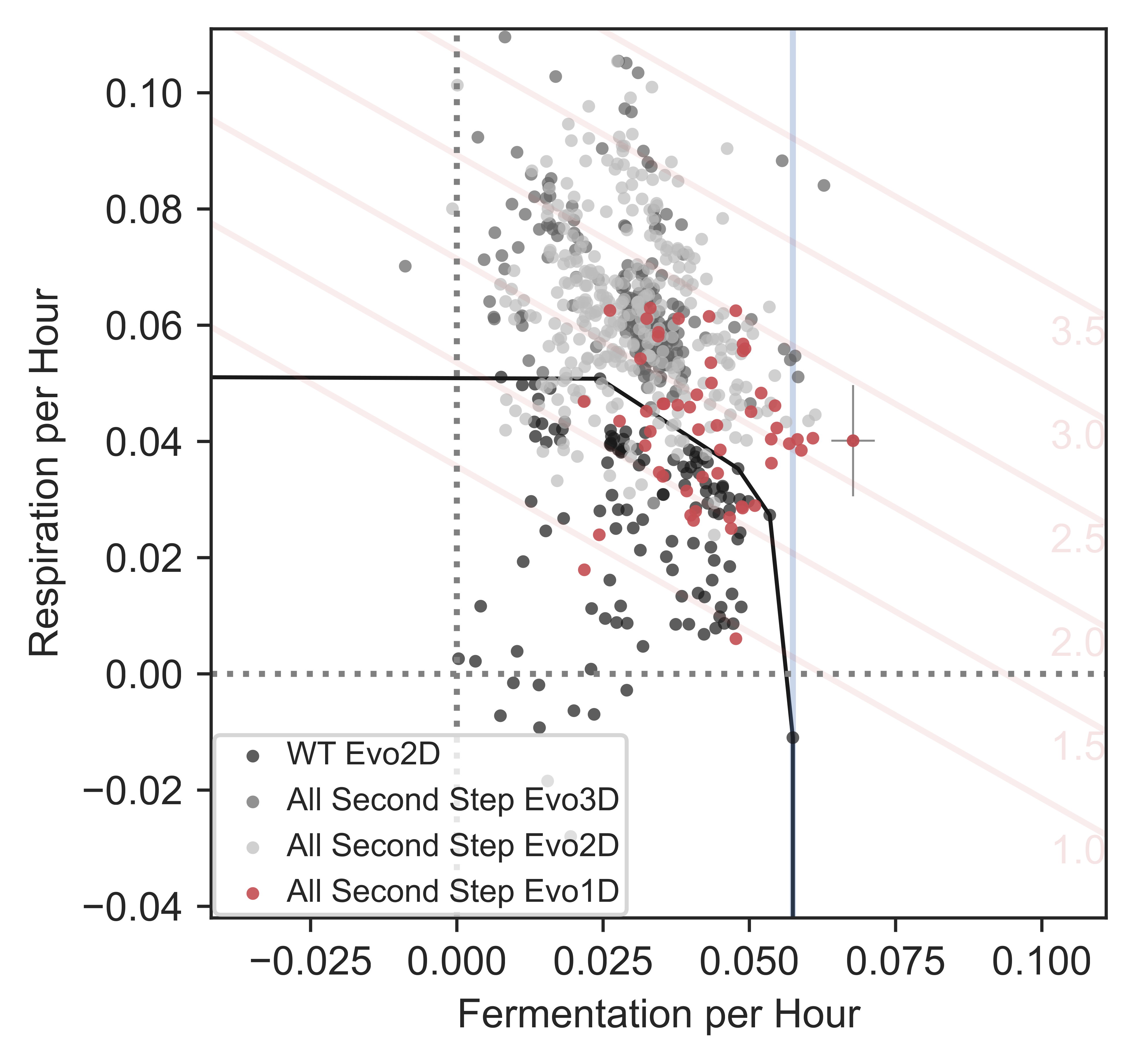

Supplement: S2 Fig — Fermentation and respiration performances for mutants discussed in the main text and Evo1D mutants (in red). Despite less dense sampling, we find at least 1 Evo1D mutant (indicated with red arrow) with fermentation performance that exceeds the highest fermentation performance from first-step mutants (blue vertical line). Error bars on mutant with high fermentation performance denote 2 standard deviations of measurement error. The data and code underlying this figure can be found in https://zenodo.org/records/13336585. (TIF) [file pbio.3002848.s002.tif]

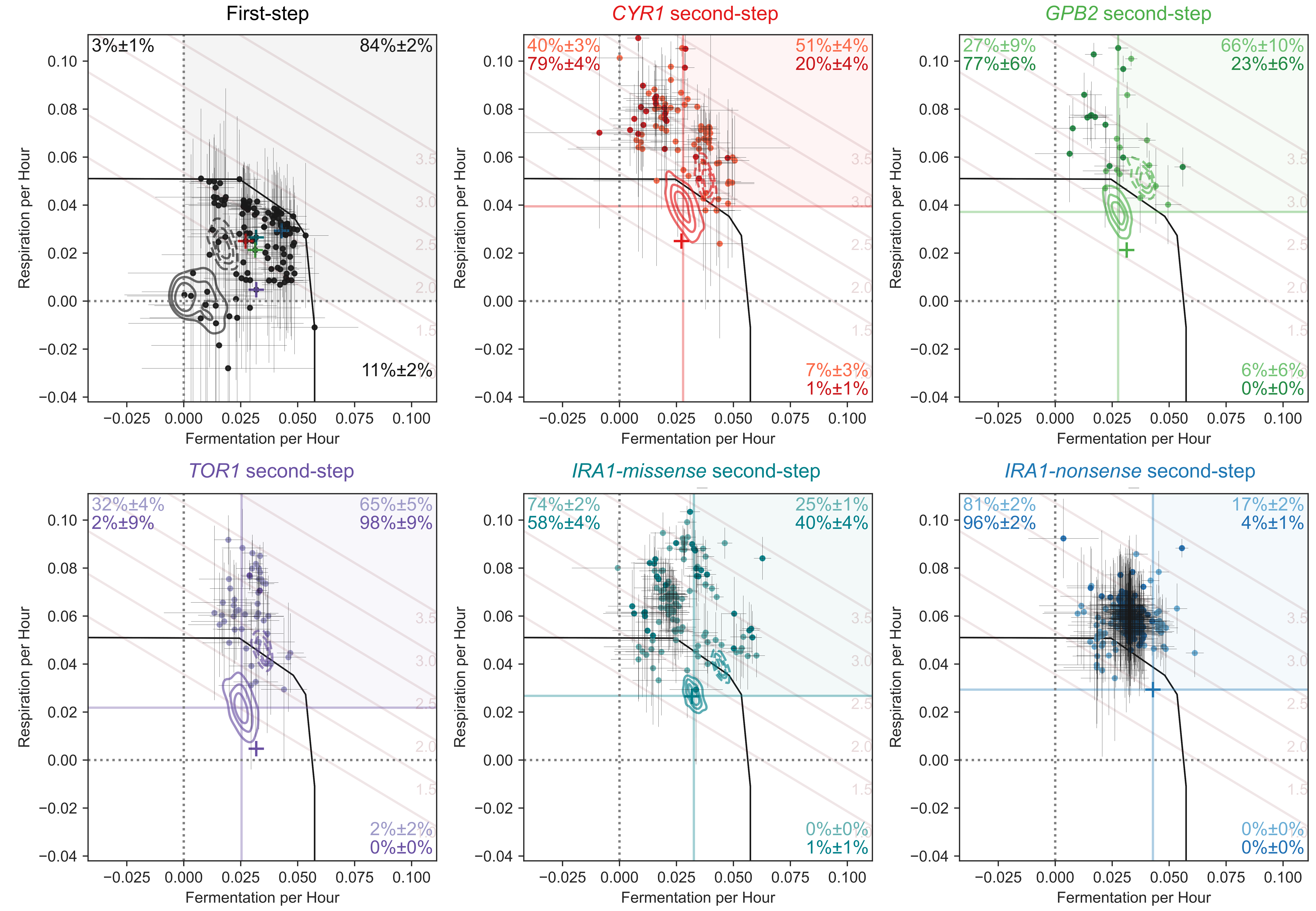

Supplement: S3 Fig — Each subpanel depicts a scatter plot with the fermentation and fermentation performances for each parental strain as in Fig 3. Lighter points indicate Evo2D mutants, darker points indicate Evo3D mutants. Error bars denote 2 standard deviations of measurement error per point. The data and code underlying this figure can be found in https://zenodo.org/records/13336585. (TIF) [file pbio.3002848.s003.tif]

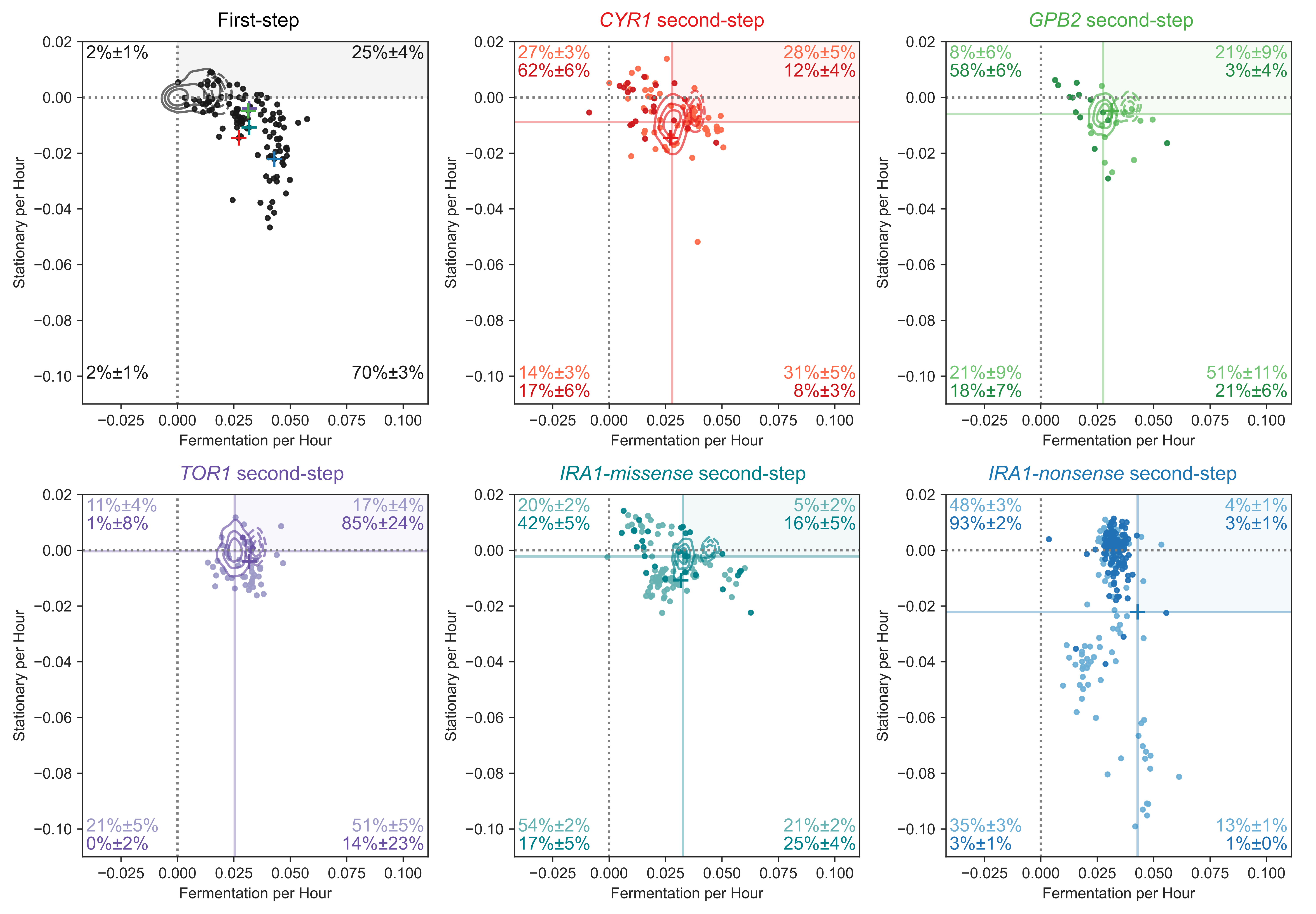

Supplement: S4 Fig — Each subpanel depicts a scatter plot with the fermentation and stationary performances for each parental strain. Lighter points indicate Evo2D mutants, darker points indicate Evo3D mutants. The data and code underlying this figure can be found in https://zenodo.org/records/13336585. (TIF) [file pbio.3002848.s004.tif]

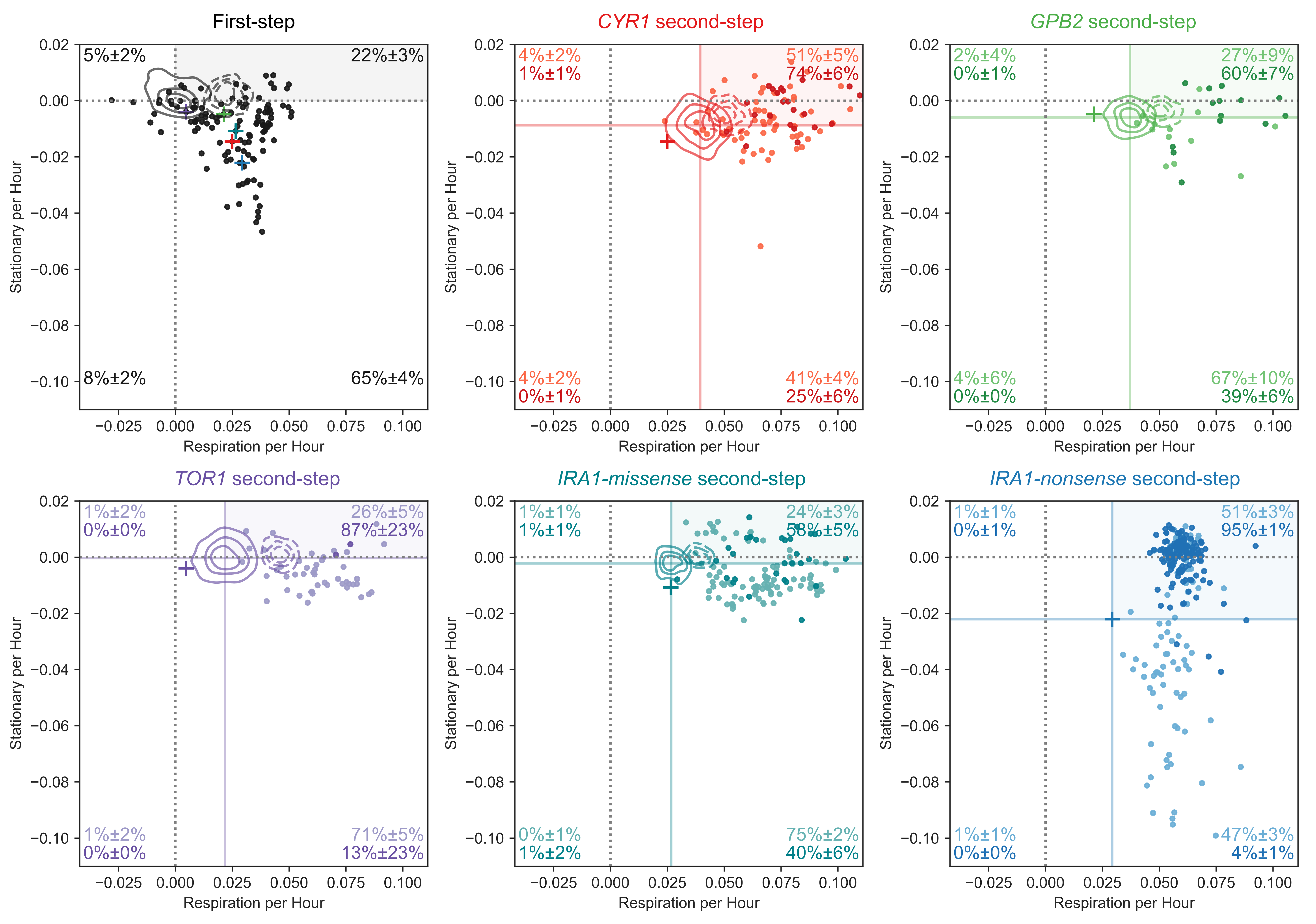

Supplement: S5 Fig — Each subpanel depicts a scatter plot with the respiration and stationary performances for each parental strain. Lighter points indicate Evo2D mutants, darker points indicate Evo3D mutants. The data and code underlying this figure can be found in https://zenodo.org/records/13336585. (TIF) [file pbio.3002848.s005.tif]

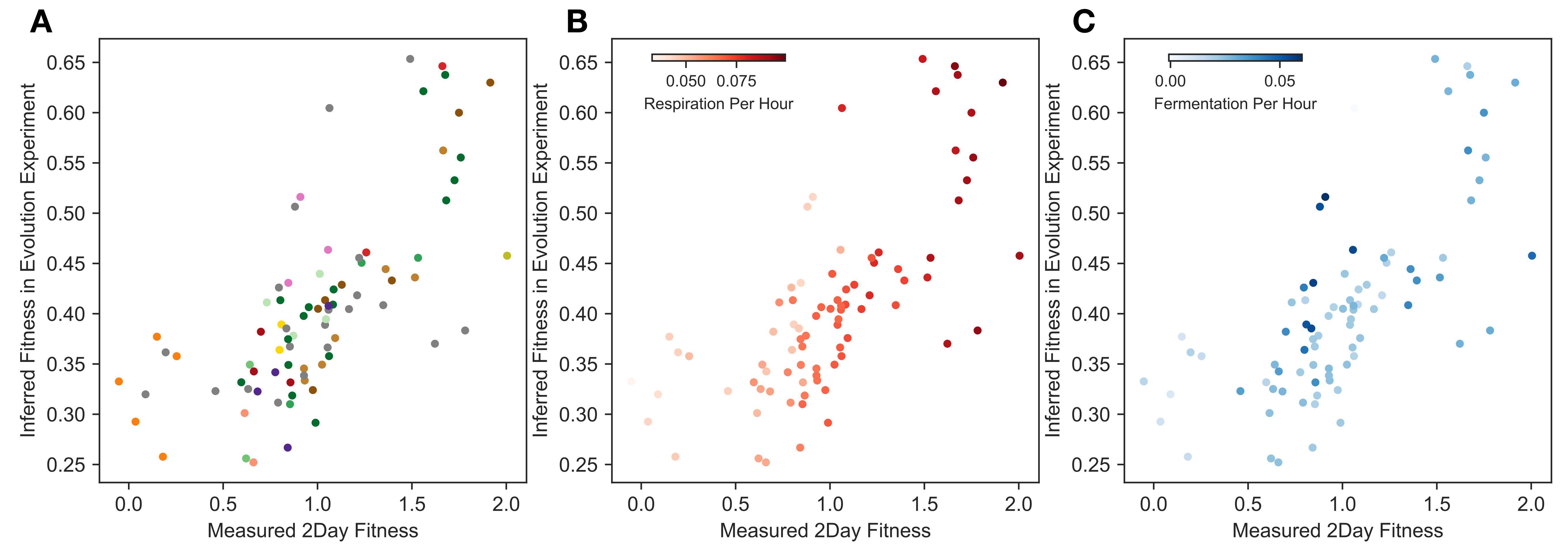

Supplement: S6 Fig — Each subpanel depicts a scatter plot comparing the measured Fit2D fitness on the x-axis and the estimated fitness from the evolution trajectories for Evo2D IRA1-missense mutants. (A) Mutants colored by gene. (B) Mutants colored by respiration performance. (C) Mutants colored by fermentation performance. The data and code underlying this figure can be found in https://zenodo.org/records/13336585. (TIF) [file pbio.3002848.s006.tif]

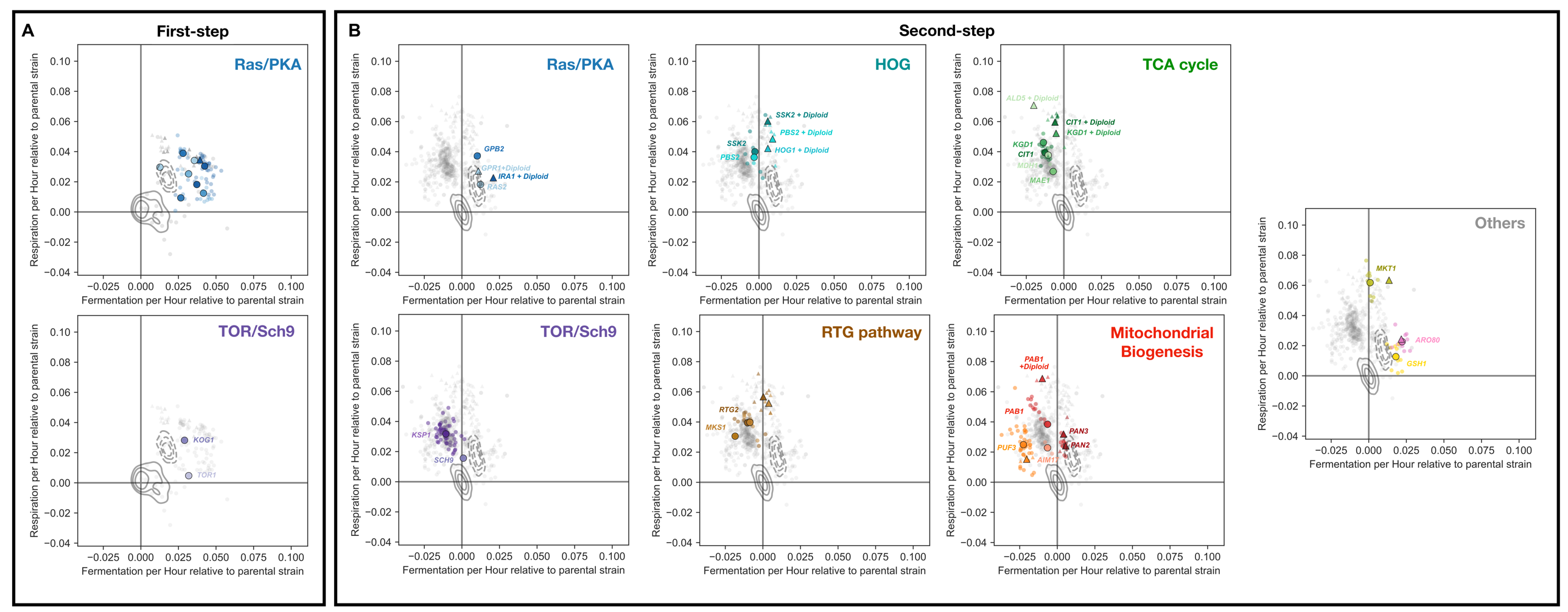

Supplement: S7 Fig — Performance effects of mutations separated by biological process or pathway as in Fig 5. Points are colored by gene, and shape indicates ploidy (circles are haploids, triangles diploids). Kernel Density Estimates show density of neutral haploids for each parental strain (solid lines) and pure diploids for each parental strain (dashed lines). (A) First-step mutants. (B) Second-step mutants depicted, with performances measured relative to parental strain. The data and code underlying this figure can be found in https://zenodo.org/records/13336585. (TIF) [file pbio.3002848.s007.tif]

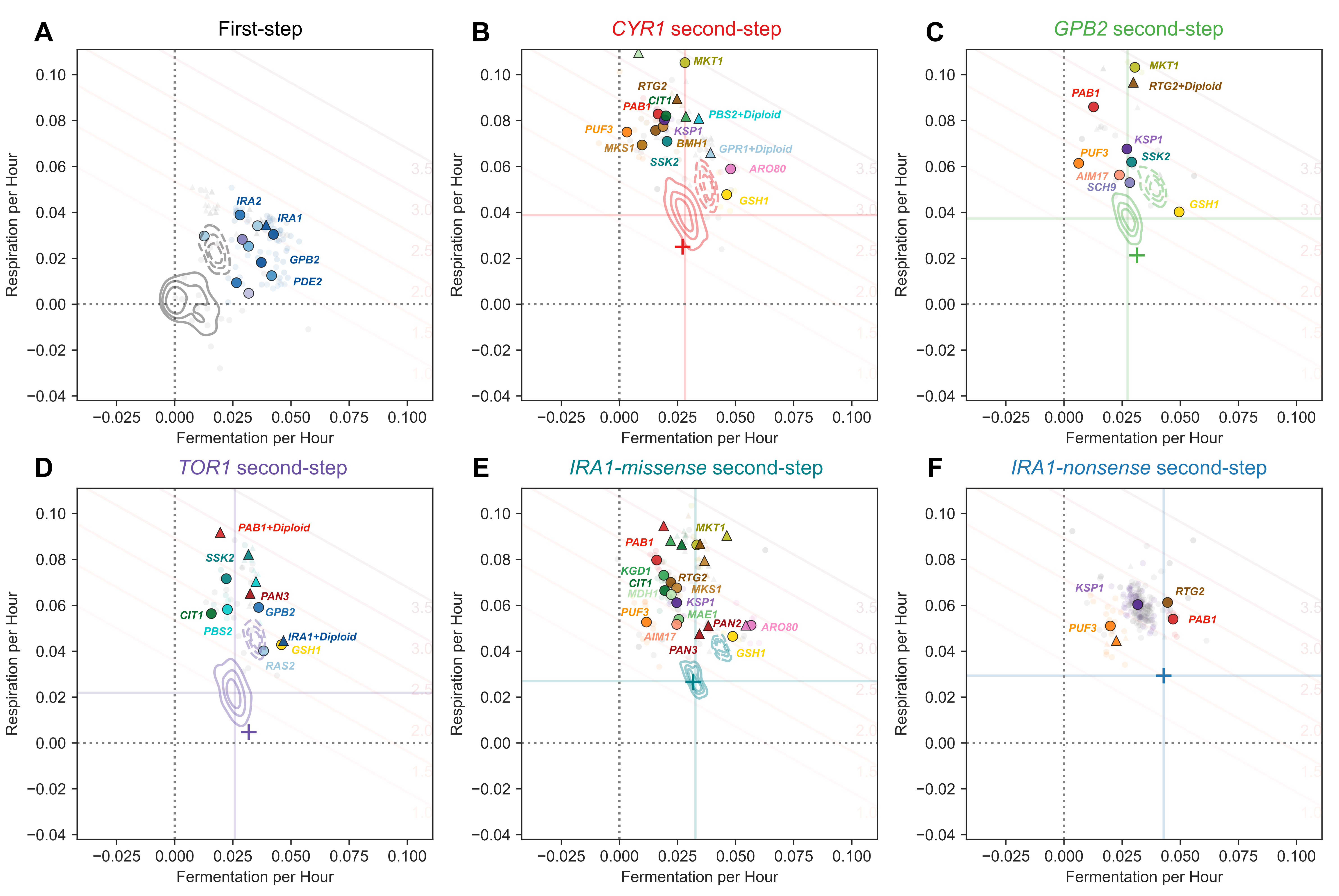

Supplement: S8 Fig — Colored by gene, shape depicts ploidy (circles are haploids, triangles diploids). Kernel Density Estimates show density of neutral haploids for each parental strain (solid lines) and pure diploids for each parental strain (dashed lines). The data and code underlying this figure can be found in https://zenodo.org/records/13336585. (TIF) [file pbio.3002848.s008.tif]

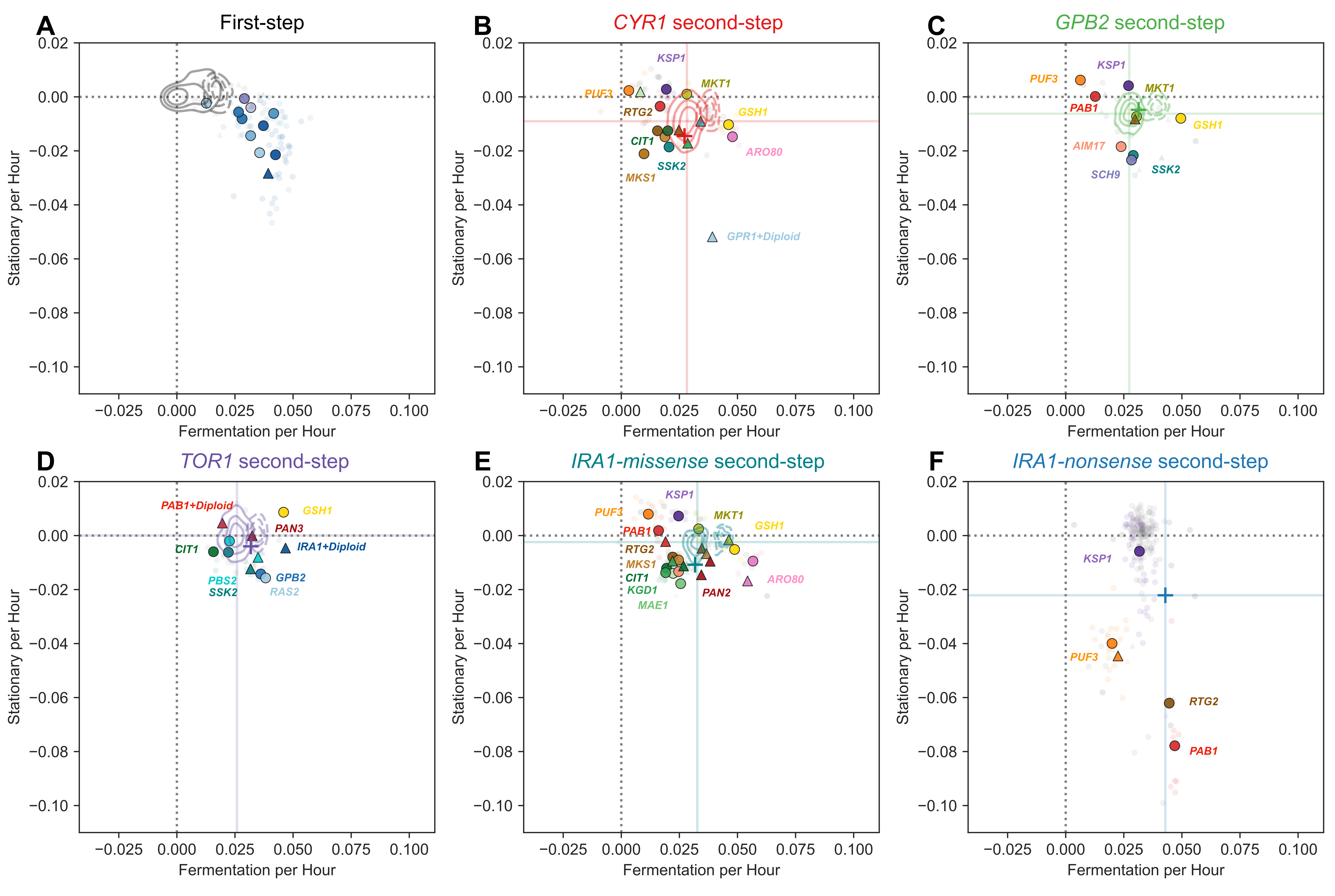

Supplement: S9 Fig — Colored by gene, shape depicts ploidy (circles are haploids, triangles diploids). Kernel Density Estimates show density of neutral haploids for each parental strain (solid lines) and pure diploids for each parental strain (dashed lines). The data and code underlying this figure can be found in https://zenodo.org/records/13336585. (TIF) [file pbio.3002848.s009.tif]

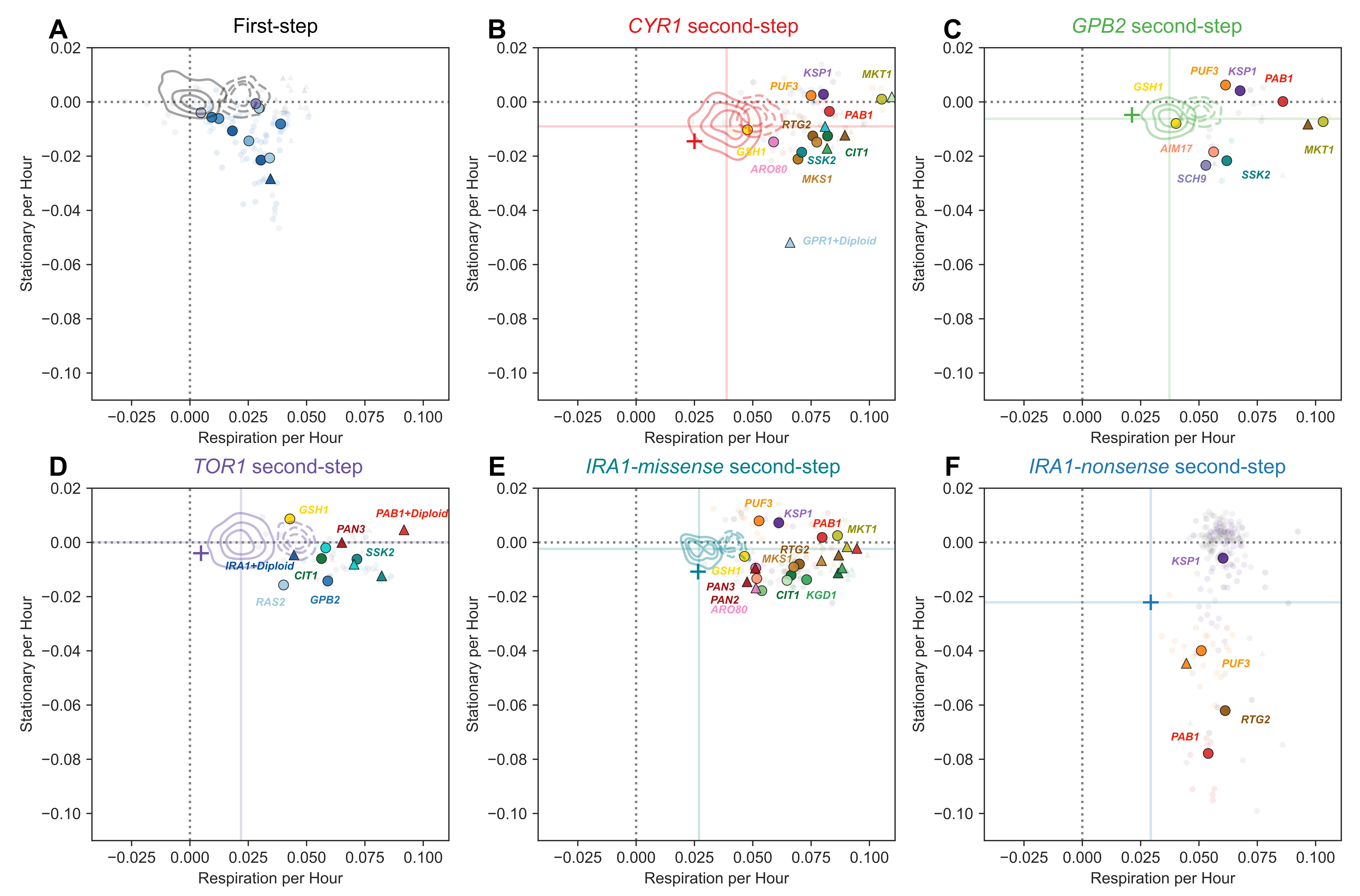

Supplement: S10 Fig — Colored by gene, shape depicts ploidy (circles are haploids, triangles diploids). Kernel Density Estimates show density of neutral haploids for each parental strain (solid lines) and pure diploids for each parental strain (dashed lines). The data and code underlying this figure can be found in https://zenodo.org/records/13336585. (TIF) [file pbio.3002848.s010.tif]
