# Supplementary material for: A high-resolution two-step evolution experiment in yeast reveals a shift from pleiotropic to modular adaptation
Source: PLoS Biol. 2024 Dec 5;22(12):e3002848. doi: 10.1371/journal.pbio.3002848 (PMC11620474; doi:10.1371/journal.pbio.3002848)
Supplement: Attachment — Submitted filename: RESPONSE TO REVIEWERS.docx [file pbio.3002848.s014.docx]

We thank the reviewers and the editor for their time, effort and constructive input. We have modified the manuscript in response to their suggestions. Below, our point by point responses are indicated with yellow highlighting, while the reviewers’ and editor’s comments are italicized.

*Dear Grant,*

*Thank you for your patience while your manuscript "A shift from pleiotropic to modular adaptation revealed by a high-resolution two-step adaptive walk" went through peer-review at PLOS Biology. Your manuscript has now been evaluated by the PLOS Biology editors, an Academic Editor with relevant expertise, and by three independent reviewers.*

*You’ll see that reviewer #1 says that he enjoyed the manuscript, but raises two issues with the interpretation and implications; one is about the framing around the “cost of complexity” hypothesis, and the other is the generalisability of the findings beyond the system under study. He has a number of other textual and/or presentational points. Reviewer #2 likes the study and the paper, but asks about a potential confound and requests several clarifications. Reviewer #3 is also positive, and really only has one significant question, which is related to reviewer #1’s point about generalisability, and draws on lessons learned from the LTEE. We discussed these comments with the Academic Editor, who also asked me to pass on some additional guidance to you (see foot of this email).*

*In light of the reviews, which you will find at the end of this email, we are pleased to offer you the opportunity to address the comments from the reviewers in a revision that we anticipate should not take you very long. We will then assess your revised manuscript and your response to the reviewers' comments with our Academic Editor aiming to avoid further rounds of peer-review, although might need to consult with the reviewers, depending on the nature of the revisions.*

*IMPORTANT:*

*a) Please change your title to something more explicit and engaging for our broader readership. We suggest something like: "A high-resolution two-step evolution experiment in yeast shows that mutations shift from pleiotropic to modular adaptation"*

We have changed the title to:

“A high-resolution two-step evolution experiment in yeast reveals a shift from pleiotropic to modular adaptation”

*b) Please address the concerns raised by the reviewers, AND the additional comments from the Academic Editor (see foot of this email).*

We have done so.

*c) Please ensure that you comply with PLOS' Data Policy; specifically, we need you to supply the numerical values underlying Figs 2DEF, 3ABC, 4AB, 5ABC, S1ABCD, S2, S3, S4, S6, S6ABCDEF, S7ABCDEF, S8ABCDEF, either as a supplementary data file or as a permanent DOI’d deposition. I note that you already have an associated GitHub deposition and a raw-looking supplementary data file. Please ensure that the data and code required to generate the Figs are made available. Also, because Github depositions can be readily changed or deleted, please make a permanent DOI’d copy (e.g. in Zenodo) and provide this URL (see below).*

We have uploaded the data as supplemental files.

S1 Data: performances by mutant

S2 Data: mutation identification details

S3 Data: comparison with evolution condition

Additional data and code are available on Zenodo:

https://zenodo.org/records/13336585

*d) Please cite the location of the data clearly in all relevant main and supplementary Figure legends, e.g. “The data underlying this Figure can be found in S1 Data” or “The data underlying this Figure can be found in* [*https://zenodo.org/records/XXXXXXXX*](https://zenodo.org/records/XXXXXXXX)

Done.

*e) Please make any custom code available, either as a supplementary file or as part of your data deposition.*

Custom code has been made available in the DOI’d version of the Github repository:

https://zenodo.org/records/13336585

*We expect to receive your revised manuscript within 1 month. Please email us (plosbiology@plos.org) if you have any questions or concerns, or would like to request an extension.*

*At this stage, your manuscript remains formally under active consideration at our journal; please notify us by email if you do not intend to submit a revision so that we withdraw the manuscript.*

***IMPORTANT - SUBMITTING YOUR REVISION***

*Your revisions should address the specific points made by each reviewer. Please submit the following files along with your revised manuscript:*

*1. A 'Response to Reviewers' file - this should detail your responses to the editorial requests, present a point-by-point response to all of the reviewers' comments, and indicate the changes made to the manuscript.*

**NOTE: In your point-by-point response to the reviewers, please provide the full context of each review. Do not selectively quote paragraphs or sentences to reply to. The entire set of reviewer comments should be present in full and each specific point should be responded to individually.*

*You should also cite any additional relevant literature that has been published since the original submission and mention any additional citations in your response.*

*2. In addition to a clean copy of the manuscript, please also upload a 'track-changes' version of your manuscript that specifies the edits made. This should be uploaded as a "Revised Article with Changes Highlighted " file type.*

**Resubmission Checklist**

*When you are ready to resubmit your revised manuscript, please refer to this resubmission checklist:* [*https://plos.io/Biology_Checklist*](https://plos.io/Biology_Checklist)

*To submit a revised version of your manuscript, please go to* [*https://www.editorialmanager.com/pbiology/*](https://www.editorialmanager.com/pbiology/) *and log in as an Author. Click the link labelled 'Submissions Needing Revision' where you will find your submission record.*

*Please make sure to read the following important policies and guidelines while preparing your revision:*

**Published Peer Review**

*Please note while forming your response, if your article is accepted, you may have the opportunity to make the peer review history publicly available. The record will include editor decision letters (with reviews) and your responses to reviewer comments. If eligible, we will contact you to opt in or out. Please see here for more details:*

[*https://blogs.plos.org/plos/2019/05/plos-journals-now-open-for-published-peer-review/*](https://blogs.plos.org/plos/2019/05/plos-journals-now-open-for-published-peer-review/)

**PLOS Data Policy**

*Please note that as a condition of publication PLOS' data policy (*[*http://journals.plos.org/plosbiology/s/data-availability*](http://journals.plos.org/plosbiology/s/data-availability)*) requires that you make available all data used to draw the conclusions arrived at in your manuscript. If you have not already done so, you must include any data used in your manuscript either in appropriate repositories, within the body of the manuscript, or as supporting information (N.B. this includes any numerical values that were used to generate graphs, histograms etc.). For an example see here:* [*http://www.plosbiology.org/article/info%3Adoi%2F10.1371%2Fjournal.pbio.1001908#s5*](http://www.plosbiology.org/article/info%3Adoi%2F10.1371%2Fjournal.pbio.1001908#s5)

**Blot and Gel Data Policy**

*We require the original, uncropped and minimally adjusted images supporting all blot and gel results reported in an article's figures or Supporting Information files. We will require these files before a manuscript can be accepted so please prepare them now, if you have not already uploaded them. Please carefully read our guidelines for how to prepare and upload this data:* [*https://journals.plos.org/plosbiology/s/figures#loc-blot-and-gel-reporting-requirements*](https://journals.plos.org/plosbiology/s/figures#loc-blot-and-gel-reporting-requirements)

**Protocols deposition**

*To enhance the reproducibility of your results, we recommend that if applicable you deposit your laboratory protocols in* [*protocols.io*](http://protocols.io/)*, where a protocol can be assigned its own identifier (DOI) such that it can be cited independently in the future. Additionally, PLOS ONE offers an option for publishing peer-reviewed Lab Protocol articles, which describe protocols hosted on* [*protocols.io*](http://protocols.io/)*. Read more information on sharing protocols at* [*https://plos.org/protocols?utm_medium=editorial-email&utm_source=authorletters&utm_campaign=protocols*](https://plos.org/protocols?utm_medium=editorial-email&utm_source=authorletters&utm_campaign=protocols)

*REVIEWERS' COMMENTS:*

*Reviewer #1:*

*[identifies himself as Alex Couce]*

*In this manuscript, Kinsler and cols. couple experimental evolution with high-resolution barcode tracking to study pleiotropy patterns among adaptive mutations in a yeast under glucose-limited, batch culture conditions. To break down fitness gains into individual trait improvements (thus quantifying pleiotropy), they leverage the specific physiology of yeast in these conditions, in which they experience two sequential growth phases: first, fermenting glucose to ethanol, and then respiring the ethanol produced in the previous phase. This growth cycle, combined with high-resolution barcode tracking, allows for a detailed analysis of pleiotropy, both in terms of abundance and sign (i.e., whether improvements in one phase are correlated, anticorrelated, or show no correlation with the other phase). In a prior work, the authors tracked the frequency changes of hundreds of mutants generated in the ancestral strain across both the fermentative and respiratory phases, revealing that the vast majority of the mutants improved performance in both phases, not just in one. Here, they conduct a follow-up evolution experiment to examine the distribution of pleiotropic effects among adaptive mutations generated in five different backgrounds, each carrying a single beneficial mutation acquired in the prior work. In contrast to their observations with the ancestral strain, they report that in this second adaptive step, most of the new beneficial mutations improve performance in only a single growth phase (respiration). Consistent with this drop in the abundance of adaptively pleiotropic mutations, they show that the molecular targets of selection also shift from glucose-sensing pathways to targets related to mitochondrial function and respiration. They argue that this shift from pleiotropic to modular phenotypic effects may be a general phenomenon during the initial steps of adaptation to novel selective pressures. In particular, they hypothesize that sensing pathways might have been shaped during past evolutionary history to coherently affect multiple traits at once, especially traits important for handling concomitant selective pressures.*

*Overall, I enjoyed the manuscript. The questions posed are relevant, and their methodology provides a uniquely sound way of exploring them. I only have two issues that have to do with the interpretation of their findings and their implications within the broader context of the field. First, the way the manuscript is framed from the start (opening of the abstract and the introduction), it reads as if it is going to be a rebuttal of Orr's 2000 "cost of complexity" hypothesis, the reduction in adaptation rate imposed by pleiotropy. This is problematic because, for one thing, later work predicts that such cost should either be much less substantial or even absent (Wang, PNAS 2010, PMID: 20876104; Wagner, Nature 2008, PMID: 18368117). In addition, and even leaving a proper account of the theory aside, I think it is important to emphasize that the present work's results indicate that a core assumption of Orr's model — that pleiotropy is forcibly random — is inaccurate, rather than suggesting that Orr's predictions are incorrect. In fact, the shift to non-pleiotropic and small-effect adaptive mutations can be seen as consistent with these predictions. I'd encourage the authors to elaborate more in detail how the 'cost of complexity' hypothesis and its implications are affected by their findings.*

We thank the reviewer for their thoughtful review and comments.

We thank the reviewer for these comments regarding the framing of our work in regards to the cost of complexity hypothesis. We do indeed agree that one of the strengths of our work is highlighting the nature of pleiotropic adaptation and that the notion that pleiotropy is “random” fails to capture the reality of the structure of pathways and regulation that can be exploited in these evolution experiments.

To address the reviewer’s comments, we have included references to the work suggested above as well as some additional text to emphasize other empirical work that demonstrates that pleiotropy is not necessarily costly:

*“Despite these theoretical expectations, empirical evidence suggests that mutations are not necessarily modular nor small in their effect on fitness. Studies of segregating variants in populations and those that arise in evolution experiments have found that pleiotropic variants can have large effects on traits and fitness* [*(Wagner and Zhang 2011; Wang, Liao, and Zhang 2010; Ruelens, Wynands, and de Visser 2023)*](https://paperpile.com/c/G7Hscl/DOab+eFMx+uCjI)*, though the extent to which these variants improve, rather than harm, multiple traits is often not characterized.”*

We have also re-written a later paragraph in the introduction to reference the notion of “pleiotropic modularity/variational modularity” (though we refrained from using this language explicitly as we thought it would confuse the reader):

*“How do we reconcile our observations of pleiotropic adaptation* [*(Y. Li et al. 2018)*](https://paperpile.com/c/G7Hscl/vIqB) *with theoretical expectations that these large-benefit mutations should affect only a small number of traits? One possibility is that fermentation and respiration performances are not as distinct as we believe and that these two modes of metabolism belong to the same physiological module* [*(Wagner and Zhang 2011; Wagner, Pavlicev, and Cheverud 2007; Wang, Liao, and Zhang 2010)*](https://paperpile.com/c/G7Hscl/DOab+lzrj+eFMx)*. However, these growth phases have distinct transcriptional, proteomic, and metabolomic profiles* [*(DeRisi, Iyer, and Brown 1997; Schlossarek et al. 2022; Zampar et al. 2013; Murphy et al. 2015)*](https://paperpile.com/c/G7Hscl/1qVP+pkhf+TwF4+cshN)*, and, moreover, a number of adaptive mutations in our series of yeast experiments do improve only one of these performances, demonstrating that it is in fact possible to shift one performance without affecting the other.”*

We also reflect on this shift from thinking about pleiotropy as “random” effects to coherence in the final paragraph of discussion:

*“Finally, we believe that the existence of these high-leverage pleiotropic routes of adaptation must be incorporated into our thinking of the evolution of complex systems. Indeed, we commonly think of pleiotropy as purely random, with mutations shifting multiple traits at once in a random and thus largely incoherent way. This generates expectations that pleiotropy should be costly, as such incoherent shifts lead to a generically disorganized state. Given that actual organisms have low-dimensional but pleiotropic signaling and regulatory systems* [*(Wagner and Zhang 2011)*](https://paperpile.com/c/G7Hscl/DOab)*, pleiotropy can often be coherent and thus might often enhance adaptive potential and allow for surprisingly large-effect adaptive mutations. It is therefore important to think of regulation and adaptation as two sides of the same problem of how to change complex and tightly integrated systems in an adaptive manner.”*

*Second, while I like the idea that signaling pathways may serve as high-leverage routes for improving many traits simultaneously, I do not think at this point we have enough evidence to determine whether this is a general phenomenon or, in contrast, whether it is just a peculiarity of Ras/PKA mutations in yeast undergoing two consecutive growth phases in glucose-limited batch cultures. In fact, the other example of a first-step mutation involving a signaling pathway shown by the authors is clearly modular (TOR/Sch9a), improving fermentation but not respiration. To be clear, I find the idea plausible, and in fact I believe it has been partly anticipated in earlier suggestions that adaptive pleiotropy could emerge due to selection on co-varying traits (Cheverud 1996,* [*doi.org/10.1093/icb/36.1.44*](http://doi.org/10.1093/icb/36.1.44)*). However, I missed a discussion on the prevalence of adaptive pleiotropy examples in other model systems. For instance, in Lenski's Long Term Evolution, it is known that the two primary traits under selection are the shortening the lag phase and the increase of the exponential growth rate (Vasi 1994,* [*doi.org/10.1086/285685*](http://doi.org/10.1086/285685)*). Several large effect mutations appear in parallel across the replicate populations in the first step of adaptation, including mutations in regulatory genes (fis, nadR, spoT, topA). Of these, only spoT seems to be a good candidate for pleiotropically improving the two traits under selection, while others are presumably much more modular, improving only growth rates (e.g. topA) (Cooper, 2003; PMID: 12538876). I think the greater value of the present work lies not in suggesting that adaptive pleiotropy may be general, but in demonstrating that it is more common than previously anticipated. As a consequence, I believe that explicitly stating the knowledge gap in the prevalence of adaptive pleiotropy, rather than (or in addition to) just speculating about its presumed generality in signaling networks, will enhance the overall impact of the work by encouraging others to explore these questions in their model systems.*

Thank you for this comment. We also hope that our study can inspire further investigation into pleiotropic adaptation, its timescale, and mechanisms. We have added the following paragraph to the discussion:

*“The extent to which pleiotropic adaptation drives early evolution in other systems remains to be determined. A recent meta-analysis of evolution experiments in E. coli found that single nucleotide variants (but not indels or structural variants) in genes with many interactions do tend to provide larger fitness benefits than those with fewer protein-protein interactions* [*(Ruelens, Wynands, and de Visser 2023)*](https://paperpile.com/c/G7Hscl/uCjI)*. In the E. coli long-term evolution experiment (LTEE), spoT mutations arise early in several replicate populations and have been shown to both improve growth rate and ability to exit lag phase* [*(Cooper, Rozen, and Lenski 2003)*](https://paperpile.com/c/G7Hscl/enpg)*. However, there are other mutations (e.g., in hslU and pykF) that arise early across parallel LTEE replicates without clear links to improving multiple traits* [*(Good et al. 2017)*](https://paperpile.com/c/G7Hscl/v0Fk)*. We hope that our study motivates further investigation into the prevalence of pleiotropic adaptation and its underlying mechanistic basis in other systems.”*

We have additionally tempered language throughout the paper to reflect the lack of robust general results across systems.

*Minor points:*

*- Abstract: "Our results suggest that the genes in cellular signaling pathways are particularly capable of providing large, adaptively pleiotropic benefits to the organism due to their ability to coherently affect many phenotypes at once." <- As explained above, at this point, with only one example in favor (Ras/PKA) and one against (TOR/Sch9a), this statement comes across as too strong. Consider phrasing it to better reflect the hypothetical nature of the idea: "_may be more likely to provide_ large, adaptively pleiotropic benefits"*

Agreed. We’ve changed the sentence as suggested. It now reads:

“Our results suggest that the genes in cellular signaling pathways may be more likely to provide large, adaptively pleiotropic benefits to the organism due to their ability to coherently affect many phenotypes at once.”

*- Figure 2C: Since much of the discussion is about whether adaptive pleiotropy is a general property of signaling systems, it'd be useful to clarify here that four of the five first-step mutations involved the Ras/PKA signaling patwhay (CYR1, GPB2, both IRA1), while the remainder involves the TOR/Sch9 one. Alternatively, this may be indicated on the upper side of Table 1.*

We have added the pathway to the top of the table.

We also have changed the title of this section to specifically refer to reduction of targets in Ras/PKA:

## *“The reduction of mutational targets in the Ras/PKA pathway drives the shift towards modular adaptation”*

*- Fig 3C: I missed an explanation in the legend about the meaning of the red arrows, as well as a reminder that, compared to panel B, we are now looking at values relative to the ancestor. Also, please spell out "KDE" in "KDE estimates".*

We have added a caption in the legend to clarify the meaning behind the red arrows:

“*Red arrows indicate very fit clones that improve both fermentation and respiration performances which have acquired three or four adaptive mutations.*”

We have also spelled out Kernel Density Estimates in each location where it was used.

*L130-132: "This suggests that these populations have not yet reached physiological constraints but rather that adaptive walks may be constrained by genetic modules which prevent adaptive mutations from improving multiple performances in a single step". I got stuck in this convoluted sentence and had to read the whole paper to grasp its meaning (i.e., that genetic constraints matters more than physiological constraints?). Please consider rewriting, it could be perfectly broken down into 2-3 sentences introducing and explaining the different concepts involved.*

Thank you for this suggestion. We have re-written this sentence:

*“This suggests that these populations have not yet reached physiological constraints, which prevent further improvement of the growth phases measured here. Instead, these data suggest that adaptation may be constrained by the structure of genetic modules which prevent adaptive mutations from improving multiple performances in a single step.”*

*L205: I think it'd be helpful to explicitly state here that the ancestral strain is not barcoded and represents >90%.*

We changed the text to more explicitly state the ancestral fraction. The text now reads:

“*We then mixed this pool of barcoded yeast with a set of barcoded neutral lineages and the ancestral strain, such that the barcoded pool started at either 2% or 5% frequency in the population, the neutral barcoded lineages collectively represented 2% of the population, and the ancestral strain made up the remaining >90% of the population*”

We note that the ancestral strain is actually barcoded, though with a barcode sequence that we digest out during library preparation. We felt this was too detailed for the main text and so it is in the methods section listed: “Restriction digest of ancestral strain’s barcode”.

*L237: "selection coefficients per cycle". Please consider giving at least an intuition of number of generations per cycle to allow comparisons with other experimental systems.*

Thank you for this suggestion. We have done so:

*“with adaptive mutations that provide fitness benefits of 1.0 or greater (per cycle; ~8 generations per cycle)”*

*L262: "fitness benefits of 55%, 48%, and 52%". Are these differences significant?*

They are indeed. We have now included the standard error of these estimates in the text:

*“fitness benefits of 55% (±0.7%), 48% (±0.7%), and 52% (±0.8%) per cycle to CYR1, GPB2, and IRA1-missense strains”*

*L302: please clarify the meaning a purpose of "re-sampling test", I'd assume it refers to the precision of the estimates?*

We thank the reviewer for pointing out that this was unclear. The purpose of the re-sampling test is to understand our degree of certainty that the fraction of mutants in each quadrant has changed between the first- and second- steps. We’ve clarified the language in the main text:

“*We found that while 85% (±3% by resampling with measurement error; see Methods)...*”

And:

“*only 35% (±1%) (p<0.001 for percentage lower than first-step according to a re-sampling test; see Methods)*”

We also added a paragraph in the methods section more carefully explaining the procedure for the test:

“*To calculate the uncertainty in the proportion of mutants that improve fermentation and respiration or other performances as depicted in Figure 3C, we use a re-sampling test based on the uncertainty of performances derived from measurement error above. For example, to calculate the uncertainty in the percentage of mutants from the second-step that improve both fermentation and respiration performance, we conducted 1,000 re-samplings of all mutant performances according to measurement uncertainty. We then computed the percentage of mutants that improved both fermentation and respiration in this resampling. We repeated this computation for each re-sampling and reported the standard deviation in the percentage of mutants that are in each quadrant.*”

*L330-31: "only a small number of mutants have fermentation performances worse than the original ancestor strain (Figure 3C, vertical black dashed line in each subplot)". Since there are no error bars or the like, please consider providing some indication of the uncertainty surrounding these values, as well as the extent to which the statement is robust.*

This statement was indeed not supported after factoring uncertainty in the fermentation performance. We’ve changed the sentence to read:

“*Note that while many of these mutants reduce fermentation performance from the initial first-step parental strains, no isolated mutants had fermentation performances that were significantly worse than the original ancestor strain*”

*L344-46: "This mutant does improve performance in both fermentation and respiration growth phases, albeit with most of its fitness gain coming from respiration". Same as above, please consider providing some indication of the uncertainty surrounding these values, as well as the extent to which the statement is robust. In both cases, I understand the plots are already complex to add error bars, but perhaps a figure could be added to the SI to show the magnitude of measurement errors.*

We have added a new supplemental figure (now Figure S3) which displays uncertainty in these measurements.

*L351: "some of these very fit clones have acquired third or fourth adaptive steps". That's pretty neat. Have you checked whether these clones are mutators?*

No known mutator mutations arose in the whole genome sequencing of these clones, but we have not explicitly measured mutation rate in any of these clones.

*L396: "42% of second-step Evo2D adaptive mutants have lower stationary performance than their parental strain". Please clarify whether this value refers only to points with measurement errors that does not overlap 0 (as later mentioned in L443-444).*

We added the word “significantly” in this sentence to clarify that this indeed refers only to mutants with measurement errors not overlapping 0:

“*42% of second-step Evo2D adaptive mutants have significantly lower stationary performance than their parental strain*.”

*L490: "we isolated at least one second-step mutation from the IRA1-nonsense population that improved fermentation performance above the highest fermentation performances achieved by first- or second-step mutations evolved in the 2-Day and 3-Day environments (see Fig S2). This suggests that while a fermentation performance maximum has not yet been reached, the pre-existing wiring of genetic and signaling pathways may be such that...". Again, please consider providing some indication of the uncertainty surrounding these values, as well as the extent to which the statement is robust.*

We modified the text to include measurements of uncertainty:

*From these experiments, we isolated at least one second-step mutation from the IRA1-nonsense population with fermentation performance of 6.7% (±0.19%) per hour, which significantly exceeds the highest fermentation performances achieved by first- or second-step mutations evolved in the 2-Day and 3-Day environments (5.7% per hour; see Fig S2). This suggests that a physiological maximum in fermentation performance has not yet been reached after the first adaptive step.*

Also in methods:

“*Using a threshold of at least 2 standard errors (which corresponds to a FDR of p<0.05) a single second-step mutant that arose in the Evo1D IRA1-nonsense population had fermentation performance that exceeded the first-step maximum.*”

We’ve also added error bars to this mutant in the supplemental figure to reflect the uncertainty of this statement.

*L493: "This suggests that while a fermentation performance...". Please consider reminding the reader that this is the third possibility you anticipated in line 463 above.*

We have re-written this section as suggested:

*“…This suggests that a physiological maximum in fermentation performance has not yet been reached after the first adaptive step.*

*Having excluded a change in selection pressure and physiological constraint as explanations for the shift towards modular adaptation, we turn to the third possibility: that cellular wiring limits the simultaneous improvement of both fermentation and respiration performances by a single mutation. In particular, the pre-existing wiring of genetic and signaling pathways may be such that, after acquiring a first mutation that improves both fermentation and respiration, it is much easier to find mutations that improve respiration performance at the cost of fermentation performance than it is to find mutations that improve both performances or even fermentation performance at the cost to respiration performance.”*

L701: "we sought to understand the frequent observation that single adaptive mutations observed in experimental evolution, especially those of large effect, can pleiotropically improve multiple distinct performances at once. This observation is puzzling because theoretical work suggests that as pleiotropy increases, large effect adaptive mutations should become less probable". -> I am not sure I agree with the way the sentence reads. For one thing, examples of pleiotropically adaptive mutations are scarce in the literature. It is true that a consistent pattern emerging from multiple studies is that the fist steps of adaptation typically involve genes encoding global regulators. And while these mutations are pleiotropic, in most cases there is no indication that this pleiotropy is adaptive in the sense of simultaneously improving several adaptive traits at once (Hindré, 2012; PMID: 22450379). I believe the major takeaway of the present work is precisely the demonstration that large effect, pleiotropically adaptive mutations exist, rather than "understanding" a phenomenon that was not even clearly demonstrated before. In addition, "puzzling" sounds rather strong to me. Please specify this refers to Orr's work, and note that later work predicts that the cost of pleiotropy should either be much less substantial or even absent (Wang, PNAS 2010, PMID: 20876104; Wagner, Nature 2008, PMID: 18368117).

We appreciate this comment on the framing of our results in the context of other work on the cost of complexity. We’ve addressed several of these concerns throughout the paper, as noted in response to this reviewer’s first concern above. We’ve additionally removed the word “frequent” from this sentence in the discussion to emphasize this point. It now reads:

*In this study, we sought to understand the observation that single adaptive mutations observed in experimental evolution, especially those of large effect, can pleiotropically improve multiple distinct performances at once.*

We also explicitly cite Orr to make it clear we are referring to predictions of that work:

*This observation is puzzling because theoretical work suggests that as pleiotropy increases, large effect adaptive mutations should become less probable [(Orr 2000)](https://paperpile.com/c/G7Hscl/86iP).*

*Reviewer #2:*

*The manuscript by Kinsler and co-authors explores the role of pleiotropy in early adaptation. While experimental evolution studies have shown that early steps in adaptation are often pleiotropic, resulting in large fitness gains, this phenomenon has not been meticulously investigated. The authors performed evolution experiments starting from five first-step mutants identified previously. They measured the fitness of hundreds of second-step mutants and compared these to the fitness effects of the first-step mutants, indicating that initial large-effect pleiotropic adaptation shifts to more modular adaptation with on average smaller, but still significant, effect sizes. The authors then go on to show that this goes along with a shift in mutational targets involved in signaling pathways towards targets involved in respiration and mitochondrial function.*

*Overall, the study addresses an important question in evolutionary biology regarding the nature of adaptive mutations and the fascinating paradox of the theoretical "cost of complexity" contrasted with the fact that we often observe pleiotropic mutations in experimental evolution. Their findings provide valuable insights into the dynamics of evolution and hold significant general interest. The paper is well-written and their results are beautifully presented.*

We thank the reviewer for their positive review of the work.

*To determine the respiration, fermentation, and stationary performance of the mutants, the authors examined the difference between 2-Day and 1-Day fitness, the first 20 hours, and the difference between 5-Day and 3-Day fitness, respectively. However, I'm concerned about the possibility of shifts in growth rates, leading to temporal changes in the transition from fermentation to respiration or respiration to the stationary phase in mutants. These shifts could potentially impact how accurately the different fitness measurements correspond to their intended growth phases. Have the authors investigated this potential confounding issue?*

The issue raised by the reviewer here is an important one. As the reviewer notes, systematic shifts in growth of the populations have the potential to alter the selective pressures and confound our finding that the populations shift from improving both fermentation and respiration performances to primarily improving respiration performance. We have addressed this concern with the partial correlation analysis as noted below (as well as re-writing this section to clarify our analysis). The re-written paragraph is as follows:

*“The first possible explanation for the shift towards modular adaptation is that the second-step of evolution was accompanied by a change in the relative contribution of fermentation and respiration growth phases to fitness in the 2-Day transfer condition. While we took care to ensure that the population sizes, transfer times, media conditions, and other details were identical to the conditions used in the first-step evolution experiment, it could be possible that differences remain. For example, the identity of the strain comprising the majority of the population in second-step evolution experiments may have shifted the selection pressures to increase the importance of respiration performance compared to the first step of evolution. To test whether there was such a shift, we compared the fitness effects of mutations in the evolution experiment itself with our fitness measurement experiments, which more closely mimic the first-step evolution experiments because the ancestral strain comprises the majority of the population. Fitness from both methods of estimation are correlated (pearson correlation 0.7), suggesting that there is not a systematic difference in selection pressure. We explicitly looked for a systematic difference in the contribution of respiration performance to fitness by conducting a partial correlation analysis. If respiration performance contributes more to evolution fitness than expected from our fitness measurement experiments, we would expect a positive partial correlation after this accounting for fitness. However, this is not the case (r=-0.02, p=0.74), indicating that the shift from pleiotropic to modular adaptation is not due to a change in selection pressure (see Methods, Differences in selection pressure do not drive shift towards modular adaptation).”*

We also expanded on this analysis in the Methods section:

*“To evaluate whether a systematic shift in selection pressure occurred during the second-step evolution experiments, we identified mutants for which we called their evolution fitness from the estimation of the distribution of fitness effects. Because many of the remaining mutants are pure diploids whose spread may be dominated by measurement noise, we removed these mutants from the list. This resulted in a set of 185 second-step mutants. We then initially performed an analysis on mutants isolated from the IRA1-missense parental strain, which had the most (82) second-step adaptive mutants identified in this manner. We found a high correlation between the fitness measured in our barcoded fitness measurement experiments and the estimated fitness from the evolution experiment (Pearson correlation of 0.7). We also plotted respiration and fermentation performances to understand whether there are systematic differences in the contribution of respiration and fermentation to evolution fitness compared to the fitness measurement experiments (Figure S6) . To explicitly test for such a systematic shift in selection pressure, we performed a partial correlation analysis. If respiration contributes more to fitness in the evolution condition, we would expect to see a correlation between respiration performance and evolution fitness, after correcting for fitness in the measurement experiment. We find no such correlation (r=-0.01, p=0.926). We similarly see no correlation between fermentation performance and evolution fitness after performing the same correction (r=0.04, p=0.715)*

*We performed a similar partial correlation analysis for the full set of 185 second-step adaptive haploids for which evolution fitnesses were estimated, finding no relationship between respiration performance and evolution fitness, accounting for fitness measurement fitness (r=-0.02, p=0.74) nor a relationship between fermentation performance and evolution fitness after accounting for fitness measurement fitness (r=0.04, p=0.613).”*

Additionally, we have added an additional supplemental figure (below) to try to show this more explicitly. For a given level of measured fitness (x-axis), we can see that there is not a systematic effect of evolution fitness according to respiration or fermentation performance (we don’t see darker red points higher at a given x-axis value; similarly, we don’t see lighter blue points higher at a given x-axis value). Partial correlation analysis shows no significant effect.

*To determine whether the shift towards modular adaptation was not induced by a simultaneous shift in the selection pressure towards enhancing respiration, the authors conducted a partial correlation between respiration performance and fitness during the evolution experiment, while adjusting for the fitness inferred from their fitness measurements. I must admit, I'm somewhat lost regarding this analysis, and a clearer explanation would greatly aid in understanding its significance. Could the authors offer further clarification on this analysis, as I find that indicating that there wasn't a shift in selection pressure pivotal to their conclusion?*

See above. We added a supplemental figure to attempt to clarify this.

*On average, how many mutations per mutant were identified in the different Evo experiments, and was this similar to the first-step Evo experiment? Were there for example on average more mutations per clone in the first step evo experiment compared to the second step experiments?*

We still detect on average ~1 casual mutation per mutant, though we did happen to sample some with multiple causal mutations as noted.

*The authors propose that the observed shift towards modular adaptation is driven by the exhaustion of mutation targets in signaling pathways. If I understand correctly, this is mainly due to the absence of second-step adaptive mutations in signaling pathways. However, the observation of second-step mutations targeting specific improvements in respiration performance does not necessarily indicate the absence of any signaling pathway targets. Rather, the effect size of the pleiotropic mutations becomes comparable smaller, because of the cost of complexity/pleiotropy. I.e. the absence of these mutations is simply due to the comparatively larger effect sizes of the respiration-specific mutations. This is supported by the Evo1D experiment, which demonstrates that improvement in fermentation is still achievable. Therefore, it seems that the targets in signaling pathways are still present, and that it's not the exhaustion but rather just the larger effect sizes of respiration targets.*

We agree with the reviewer that both the available spectrum of mutations and the fitness effect size of these mutations are important factors that contribute to the phenomenon we observe. Of course, we agree that there can be smaller effect mutations that exist to drive adaptation (though their relevance is minimal at this stage of evolution). We also agree, in principle, that there may be additional mutations in the Ras/PKA pathway that can further optimize this pathway beyond the first step. We actually observe a couple of these (i.e.. an IRA1-nonsense mutation in an IRA1-missense experiment and an IRA1 mutation in the GPB2 parental strain). To try and emphasize this point, we’ve changed the language of the section title to remove the use of the word “exhaustion” which we feel may have suggested the contrary and to include specifically the Ras/PKA pathway.

## *“The reduction of mutational targets in the Ras/PKA pathway drives the shift towards modular adaptation”*

We also address this point in the discussion by explicitly stating that they must improve both performances to a “substantial degree in an adaptive manner”:

*“This suggests that the Ras/PKA pathway is virtually unique in its ability to modulate both fermentation and respiration performances together to a substantial degree in an adaptive manner, a notion also supported by the fact that we observe the shift towards modularity adaptation already in the second adaptive step.”*

*Line 731" These results support a model of adaptation wherein early adaptation is driven by mutations of large effect that improve multiple performances at once. Then, after these mutations have become exhausted, adaptation may proceed via more modest mutations that improve performances in a stepwise manner": I don't think their results seem to support this generalized scenario. All five second-step conditions (although the IRA1 nonsense maybe to a lesser extent) still show the presence of large effect second-step mutations, comparable to top first-step effect sizes. It's important here to note that it's on average their scenario might be the case, but depending on the conditions, for example in situations with strong clonal interference, large effects mutations will preferentially be selected for.*

The reviewer is correct that the dynamics of adaptive walks may differ depending on various factors (degree of clonal interference, etc.). In the strong clonal interference case that the reviewer mentions, the effects of adaptive modularity may be even stronger, as the most adaptive mutations we observe are those that only improve respiration performance, improve respiration performance and become auto-diploids (Figure 5B), or are comprised of a series of modular steps (Figure 5C). As such, we believe the clonal interference dynamics would perhaps support our model the strongest. On the other hand, one might not see these effects in a more mutation-limited regime, where early adaptation might be more dominated by genes with higher mutation rates. We’ve added the words “typically” and “may” to better indicate that these statements may depend on some of these details:

*“These results support a model of adaptation wherein early adaptation is typically driven by mutations of large effect that improve multiple performances at once. Then, after these mutations have become exhausted, adaptation may proceed via more modest mutations that improve performances in a stepwise manner.”*

*The authors mention that they performed evolution experiments in replicate, but it is unclear how they take the resulting variation into account in their downstream analyses. Did both replicate population behave similarly?*

The replicate evolution experiments did behave more or less similarly. For example, we did compare the distribution of fitness effects between replicate conditions, finding that they were very similar to each other. One reason for performing the experiments in replicate is to increase the sampling of the number of adaptive mutations per experiment. For example, one replicate Evo2D IRA1 missense experiment became dominated by a very fit clone quite early in the experiment, which would have hindered our ability to sample a variety of adaptive mutations from this condition. As our paper primarily focuses on the properties of the adaptive mutations isolated from these experiments, rather than the dynamics of each evolution experiment, we opted to not discuss these at length.

*Minor comments:*

*The colors differentiating Evo2D and Evo3D weren't always clearly distinguishable (Fig 2EF, 3C).*

We appreciate this comment regarding the colors of Evo2D and Evo3D points. We’ve decided to keep the colors as is to maintain clarity of parental strains throughout the manuscript.

*Why aren't the crosses in Fig 3C within the neutral isoclines? Wouldn't this be the expectation?*

The reviewer is correct that, absent measurement noise or the influence of additional mutations that arose during the barcode process, the crosses denoting the parental strains should be within the neutral isoclines indicated in the figure. Indeed, for some of the parental strains (in particular *TOR1* and *GPB2*) differ from the population of neutral lineages in their respiration performances. This may be due to additional mutations picked up during the barcoding process. We’ve added a mention to these background mutations in the section:

“*To identify the likely adaptive mutations, we compared the genes across all isolated mutants from all evolution experiments and labeled genes that were hit more than three times across all mutants as putatively causal, removing mutations that were represented across all isolated mutants derived from a given parental strain, likely reflecting mutations that arose during the barcoding process*”

We’ve also added mention of this shift in the methods section as well:

*We also noticed that the “neutral haploids” for GPB2 and TOR1 parental strains differed in respiration performance from the parental strain present in the collection of first-step mutants they were originally derived from. This difference may be due to additional mutations that arose during the barcoding process. In particular, GPB2 mutants harbor an additional LPD1 downstream variant and TOR1 mutants harbor an additional MRPL10 missense variant (see Variant calling)*.

We’ve also included a supplementary data file (Data S2) which includes all called mutations in the study.

*How many generations or transfers were conducted for Evo1D and Evo3D experiments? I couldn't find this info.*

We thank the reviewer for noticing this omission. This information has now been added to the methods section:

*“Evo2D experiments were carried out for a total of 22 transfers (~176 generations; ~8 generations/transfer). Evo1D experiments were carried out for 19 transfers (~133 generations; ~7 generations/transfer). Evo3D experiments were carried out for 21 transfers (~168 generations; ~8 generations/transfer).”*

*Fig 2EF: Slanted X-labels are too small.*

We thank the reviewer for this comment. We’ve increased the size of these labels.

*Reviewer #3:*

*[identifies himself as Christopher Marx]*

*This paper tackles a key issue in terms of adaptive trajectories: is there a particular commonality to the manner in which beneficial mutations generate their fitness effects? Using a very well-characterized yeast system and a super powerful implementation of barcodes, they show rather convincingly that, in their two (or three) phase environments the first beneficial mutations showed synergistic pleiotropy with single mutations affecting both fermentation and respiration, the second mutations were decidedly more modular and changed one or the other and very rarely both. All of this is very well done and clearly written.*

*I only have one major concern: how general is this? This is less a concern about it being just one environment and one model system, as that is true for any experimental system. It is more that - as the authors state - the vast majority of first step mutations were in the Ras/PKA pathway. Does this open the door to synergistic pleiotropy in a way that is unusual? Does this underlie the "regulation then mitochondria" ordering of mutations?*

*Knowing what the field does from other systems, this is already known not to be far from universal. One of the Lenski lines first had a mutation deleting the ribose operon, topoisomerase, ppGpp synthase, a uridyltransferase, and then pyruvate kinase. The ppGpp synthase affects a global secondary messenger, but the others are not global regulators in the traditional sense (even if they do have broad effects). Numerous other adaptive trajectories are known from experimental evolution so the opportunity to do at least some degree of comparison is available, at least at the level of gene annotations.*

*I feel what is needed is simply a bit more reach to other systems and a step back from making too big of claims of generality. It may very well come to be true that the biggest effect, first step mutations do tend to have synergistic pleiotropy and by the second mutation shift to being modular, but I think much more data is needed before making too strong of a claim.*

*Christopher Marx*

Thank you for the positive review and this suggestion. We agree that the extent to which this is general may yet to be seen. Motivated by your comment and that of reviewer 1, we’ve added the following paragraph to the discussion:

*“The extent to which pleiotropic adaptation drives early evolution in other systems remains to be determined. A recent meta-analysis of evolution experiments in E. coli found that single nucleotide variants (but not indels or structural variants) in genes with many interactions do tend to provide larger fitness benefits than those with fewer protein-protein interactions* [*(Ruelens, Wynands, and de Visser 2023)*](https://paperpile.com/c/G7Hscl/uCjI)*. In the E. coli long-term evolution experiment (LTEE), spoT mutations arise early in several replicate populations and have been shown to both improve growth rate and ability to exit lag phase* [*(Cooper, Rozen, and Lenski 2003)*](https://paperpile.com/c/G7Hscl/enpg)*. However, there are other mutations (e.g., in hslU and pykF) that arise early across parallel LTEE replicates without clear links to improving multiple traits* [*(Good et al. 2017)*](https://paperpile.com/c/G7Hscl/v0Fk)*. We hope that our study motivates further investigation into the prevalence of pleiotropic adaptation and its underlying mechanistic basis in other systems.”*

We have additionally tempered language throughout the paper to reflect the lack of robust general results across systems.

*COMMENTS FROM THE ACADEMIC EDITOR (lightly edited):*

*Besides the specific questions of rev#1 and especially rev#2 about interpretation of the results, the reviewers seem to agree that the authors should be asked to (i) better acknowledge previous work on modular pleiotropy (by Wagner and others), and (ii) particularly better discuss the generality of their findings, i.e. the decreasing pleiotropy of mutations during adaptation and the role of signalling pathways. In addition to these comments by the reviewers, I think the authors should also address the possible role of mutation bias, i.e. to which extent are first-step mutations driven by high mutation rates instead of large fitness benefits?*

As noted above, we have re-written several parts of the introduction and discussion to acknowledge work on modular pleiotropy:

*“Despite these theoretical expectations, empirical evidence suggests that mutations are not necessarily modular nor small in their effect on fitness. Studies of segregating variants in populations and those that arise in evolution experiments have found that pleiotropic variants can have large effects on traits and fitness* [*(Wagner and Zhang 2011; Wang, Liao, and Zhang 2010; Ruelens, Wynands, and de Visser 2023)*](https://paperpile.com/c/G7Hscl/DOab+eFMx+uCjI)*, though the extent to which these variants improve, rather than harm, multiple traits is often not characterized.”*

We have also re-written a later paragraph in the introduction to reference the notion of “pleiotropic modularity/variational modularity” (though we refrained from using this language explicitly as we thought it would confuse the reader):

*“How do we reconcile our observations of pleiotropic adaptation* [*(Y. Li et al. 2018)*](https://paperpile.com/c/G7Hscl/vIqB) *with theoretical expectations that these large-benefit mutations should affect only a small number of traits? One possibility is that fermentation and respiration performances are not as distinct as we believe and that these two modes of metabolism belong to the same physiological module* [*(Wagner and Zhang 2011; Wagner, Pavlicev, and Cheverud 2007; Wang, Liao, and Zhang 2010)*](https://paperpile.com/c/G7Hscl/DOab+lzrj+eFMx)*. However, these growth phases have distinct transcriptional, proteomic, and metabolomic profiles* [*(DeRisi, Iyer, and Brown 1997; Schlossarek et al. 2022; Zampar et al. 2013; Murphy et al. 2015)*](https://paperpile.com/c/G7Hscl/1qVP+pkhf+TwF4+cshN)*, and, moreover, a number of adaptive mutations in our series of yeast experiments do improve only one of these performances, demonstrating that it is in fact possible to shift one performance without affecting the other.”*

We have also added an additional paragraph of the discussion to better discuss the generality of the findings:

*“The extent to which pleiotropic adaptation drives early evolution in other systems remains to be determined. A recent meta-analysis of evolution experiments in E. coli found that single nucleotide variants (but not indels or structural variants) in genes with many interactions do tend to provide larger fitness benefits than those with fewer protein-protein interactions* [*(Ruelens, Wynands, and de Visser 2023)*](https://paperpile.com/c/G7Hscl/uCjI)*. In the E. coli long-term evolution experiment (LTEE), spoT mutations arise early in several replicate populations and have been shown to both improve growth rate and ability to exit lag phase* [*(Cooper, Rozen, and Lenski 2003)*](https://paperpile.com/c/G7Hscl/enpg)*. However, there are other mutations (e.g., in hslU and pykF) that arise early across parallel LTEE replicates without clear links to improving multiple traits* [*(Good et al. 2017)*](https://paperpile.com/c/G7Hscl/v0Fk)*. We hope that our study motivates further investigation into the prevalence of pleiotropic adaptation and its underlying mechanistic basis in other systems.”*

We have additionally tempered language throughout the paper to reflect the lack of robust general results across systems.

As for mutational bias, we find it unlikely that mutations in this pathway are likely to all have higher mutation rates to drive this enrichment, as this bias would need to be true across multiple genes in the same pathway. We also note that these mutations do have the largest fitness effects observed, with the second-step mutations showing a pattern of diminishing returns epistasis, suggesting that the mutation rate towards these mutations is not so exceedingly high to outweigh the effect of other mutations. It is possible (or even likely) that differences in mutation target size contribute to the shift towards respiration-improving mutations in the second step of evolution. We discuss an estimate for this difference in target size for IRA1-missense mutations in the text:

*“Notably, haploids that harbor mutations in these genes have similar fitness in the 2-Day transfer environment to mutants with mutations in ARO80 and GSH1, which exhibit adaptive pleiotropy. Despite these similar fitnesses, there is an 8-fold increase in observed adaptively modular genetic targets than those that are adaptively pleiotropic in the IRA1-missense 2-Day evolution experiments (41 mutants in TCA and RTG with fitnesses between 2.0 and 2.5 compared to 5 in GSH1 and ARO80 for IRA1-missense).”*

We also discuss how MKT1 mutations tend to occur in the same nucleotide and that their rarity is likely due to this small target size despite providing very strong fitness benefits:

*There are also single point mutations in MKT1 which achieve very high 2-Day fitness by greatly improving respiration performance and showing little cost to fermentation performance (Figure 5B - chartreuse circles). Interestingly, all adaptive mutations in this gene occur at the same nucleotide, changing from 89A to C,G, or T. Thus, while these mutations are driven by only a single mutation, their lower frequency reflects the reduced target size compared to the other haploid mutations which have multiple targets within the gene (e.g., those in RTG pathway, TCA cycle, etc.).*

*I suggest that the authors, for their discussion of the generality of their findings, have a look at a recently published meta-analysis. Ruelens et al. 2023 (doi:10.1098/rstb.2022.005) report a meta-analysis of the pleiotropy of mutations in lab evolution experiments with E. coli, including Lenski's LTEE and 23 other studies. The analysis shows that point mutations (but not indels or large deletions or duplications, because they cannot realize the combinations of pleiotropic effects underlying these large benefits!) in highly pleiotropic genes (measured by the number of protein-protein interactions) are among the largest-benefit mutations under constant lab conditions. These findings for E. coli may therefore provide a more general explanation for the authors’ observation, that first-step mutations in yeast are more pleiotropic than later mutations – because clonal interference will promote large-effect mutations to be fixed first.*

We thank the editor for pointing us to this paper. We have incorporated it into the introduction and discussion as noted in the response above. And we agree that the abundance of the adaptive pleiotropy in early evolution is likely to be due to the fact that we tend to focus on the adaptation-driving events that are both sufficiently common and sufficiently large-benefit.
